# Lack of TRPV1 aggravates obesity-associated hypertension through the disturbance of mitochondrial Ca2+ homeostasis in brown adipose tissue

**DOI:** 10.1038/s41440-021-00842-8

**Published:** 2022-01-18

**Authors:** Li Li, Liqun Ma, Zhidan Luo, Xiao Wei, Yu Zhao, Cui Zhou, Aidi Mou, Zongshi Lu, Mei You, Chengkang He, Huan Ma, Qing Zhou, Lijuan Wang, Tingbing Cao, Yucun Gu, Peng Gao, Zhiming Zhu

**Affiliations:** 1 Center for Hypertension and Metabolic Diseases, Department of Hypertension and Endocrinology, Daping Hospital, The Army Medical University, Chongqing Institute of Hypertension, Chongqing, 400042 China; 2 The Regenerative Medicine Center, Aston Medical School, Aston University, Birmingham, UK

**Keywords:** Obesity-associated hypertension, Brown adipose tissue, Mitochondrial Ca2+, Transient receptor potential vanilloid-1, Uncoupling protein 1, Leucine zipper-EF-hand containing transmembrane protein 1

## Abstract

The combination of obesity and hypertension is associated with high morbidity and mortality; however, the mechanism underlying obesity-induced hypertension remains unclear. In this study, we detected the possible effects of TRPV1, a previously identified antihypertensive calcium (Ca^2+^) channel in adipose tissue, on the occurrence of obesity and hypertension in mice lacking UCP1, a spontaneously genetically manipulated obesity model, by generating TRPV1 and UCP1 double knockout mice. In these mice, obesity and hypertension appeared earlier and were more severe than in mice with the knockout of UCP1 or TRPV1 alone. The knockout of TRPV1 in UCP1 knockout mice further reduced functional brown adipose tissue (BAT) generation; decreased resting oxygen consumption, heat production, and locomotor activities; and was accompanied by severe mitochondrial respiratory dysfunction in BAT. Mechanistically, TRPV1, UCP1, and LETM1 acted as a complex to maintain an appropriate mitochondrial Ca^2+^ level, and TRPV1 knockout caused a compensatory increase in mitochondrial Ca^2+^ uptake via LETM1 activation. However, the compensatory response was blocked in UCP1^−/−^ mice, resulting in dramatically reduced mitochondrial Ca^2+^ uptake and higher production of ATP and oxidative stress. This study provides in vivo evidence for the critical role of BAT mitochondrial Ca^2+^ homeostasis in obesity-associated hypertension and indicates that the TRPV1/UCP1/LETM1 complex may be an alternative intervention target.

## Introduction

Obesity has become a major public health problem. It has been reported that the obesity rate among adults is increasing in most parts of the world. The World Health Organization (WHO) pointed out that with the prevalence of obesity, the double burden of communicable and noncommunicable diseases is imminent [1]. Obesity-associated hypertension has become an epidemic health problem and a major risk factor for the development of cardiovascular diseases (CVDs) [2]. The etiology of obesity-associated hypertension is extremely heterogeneous, as it is the result of increased sodium reabsorption, the activation of the renin–angiotensin–aldosterone system, endothelial dysfunction and decreased sensitivity to natriuretic peptides, as well as oxidative and inflammatory stress [2]. The abnormal production of reactive oxygen species (ROS) has been considered a common pathogenetic mechanism of cardiovascular diseases resulting from diverse risk factors, such as chronic smoking, diabetes mellitus, and metabolic syndrome [3].

Transient receptor potential vanilloid-1 (TRPV1) is generally expressed in sensory neurons, vasculature, and adipose tissues. TRPV1 is a nonselective cation channel that transduces the sensation signals of harmful heat and pain [4]. Our previous studies showed that dietary capsaicin, a specific agonist of TRPV1, triggers nitrogen oxide production in vascular endothelial cells, thus improving endothelial-dependent vasodilation to protect against hypertension [5]. In addition, TRPV1-mediated Ca^2+^ influx inhibits lipid accumulation in white adipose tissue (WAT) by facilitating Ca^2+^ uptake in adipocytes, thus preventing diet-induced obesity [6]. Others have indicated that the activation of TRPV1 also protects brown adipose tissue (BAT) against the whitening process and promotes WAT to transdifferentiate into a beige phenotype that contains a large number of brown adipocytes within WAT [7, 8]. However, whether the regulatory effect of TRPV1 in adipose tissues plays a role in the occurrence of obesity-related hypertension remains uninvestigated.

Abundant with mitochondria expressing uncoupling protein 1 (UCP1), BAT is primarily responsible for thermogenic energy consumption [9]. As a critical marker of BAT, the activation of UCP1 uncouples the electrochemical gradient of the inner mitochondrial membrane (IMM), which drives adenosine triphosphate (ATP) production to stimulate proton leakage and generate heat [10]. Mice lacking UCP1 become obese when they are fed in a thermoneutral environment or when they grow old [11], indicating a crucial role of mitochondrial heat production in energy homeostasis and obesity. Thus, UCP1 knockout mice could be considered a naturally occurring genetically manipulated obese mouse model in which heat production from BAT is blocked.

The capacity of mitochondria to buffer Ca^2+^ plays an essential role in regulating Ca^2+^-dependent signal transduction as well as the pathophysiology of multiple diseases [12]. Ca^2+^ entering the mitochondrial matrix promotes ATP generation by activating vital tricarboxylic acid cycle enzymes [13]. Ca^2+^ influx into mitochondria at an appropriate time turns off multiple signal transductions activated by Ca^2+^ release from the endoplasmic reticulum to maintain Ca^2+^ homeostasis. Therefore, the inflow and outflow rates of Ca^2+^ must be balanced to avoid mitochondrial dysfunction. Leucine zipper-EF-hand containing transmembrane protein 1 (LETM1) is a mitochondrial inner membrane protein that mediates mitochondrial Ca^2+^ uptake and extrusion in a gradient-dependent manner, which is important for mitochondrial Ca^2+^ homeostasis [14]. LETM1 downregulation participates in impaired insulin signaling in the adipose tissue of obese mice [15]. Thus, by acting as a critical regulator of mitochondrial Ca^2+^ homeostasis, LETM1 might also be involved in the process of adipose tissue dysfunction that results in obesity-related hypertension. However, whether there exists any relationship between TRPV1 and LETM1 that regulates both cellular Ca^2+^ homeostasis in adipose tissue and how they cooperate in the development of hypertension are still unclear.

Therefore, in this study, we crossbred TRPV1 knockout mice with UCP1 knockout mice to generate TRPV1/UCP1 double knockout mice to determine the necessity of TRPV1-mediated Ca^2+^ influx to adipocytes in the maintenance of the BAT phenotype in this obesity model and investigate the possible mechanism accounting for obesity-induced hypertension. We found that the regulatory effect of TRPV1 on LETM1 was critical to restrain obesity and related hypertension, and the knockout of TRPV1 further exacerbated obesity in UCP1 knockout mice, resulting in more severe hypertension.

## Materials and methods

### Mouse genetic models

The experiment conformed to the Guide for the Care and Use of Laboratory Animals published by the US National Institutes of Health (NIH Publication No. 85-23, revised 1996). The Experimental Animal Ethics Committee of Daping Hospital approved this study. TRPV1 knockout (TRPV1^−/−^) and UCP1 knockout (UCP1^−/−^) mice were ordered from the Jackson Laboratory (Bar Harbor, ME, USA). Heterozygous progeny were produced by mating TRPV1^−/−^ mice with UCP1^−/−^ mice (both on a C57BL/6 background), which were then mated with each other to generate homozygous TRPV1^−/−^/UCP1^−/−^ mice and wild-type littermates. Male WT, TRPV1^−/−^, UCP1^−/−^, TRPV1^−/−^/UCP1^−/−^ mice, four to six weeks old, were housed at a stable temperature (24–25 °C) and received a standard chow diet (Chow) for 10 months.

### Primary culture of brown adipocytes

Primary brown adipocytes were cultured as previously reported [16]. Brown preadipocytes dissected from the interscapular BAT of 3–4-week-old WT, TRPV1^−/−^, UCP1^−/−^, TRPV1^−/−^/UCP1^−/−^ mice were digested with collagenase. Suspended cells were collected via nylon cell filters (250- and 60-µm mesh) to pellet the stromal vascular cell fraction. The cells were suspended again in DMEM after centrifugation (5 min × 800 g). During the first 3 days of induced differentiation, the differentiation media was supplemented with 20 nM insulin and 2 nM triiodothyronine. On the 4th day, the induction media was supplemented with 20 nM insulin, 2 nM triiodothyronine, 0.5 mM 3-isobutyl-1-methylxanthine and 0.5 µM dexamethasone. On the 7th day, the differentiation media was filled for 3–4 days.

### High-resolution respirometry of mitochondria in BAT

The mitochondrial respiration rate was measured with 1.0 mg of mitochondria in a high-resolution respirometry chamber filled with 2 ml MiR05 by Oxygraph-2k (Oroboros, Innsbruck, Austria) [17]. Two mitochondrial substrate–uncoupler–inhibitor titration protocols were used. The capability of complex I oxidative phosphorylation (CI OXPHOS) was determined through the addition of glutamate (5 mM) and malate (2 mM) in the presence of saturated ADP (5 mM). Succinate (10 mM) was then added to establish maximal OXPHOS capability with convergent input through complex I and II (CI + II OXPHOS). Uncoupler FCCP was subsequently titrated (1–1.5 μM each step) to determine the maximal oxygen consumption rates of complexes I + II (CI + II ETS, ETS). Residual oxygen consumption was measured by adding rotenone (0.5 μM) and antimycin A (2.5 μM).

### ROS detection

Cytosol superoxide anion was determined by dihydroetorphine hydrochloride (DHE) (Sigma, USA), and mitochondrial superoxide anion was measured by MitoSOX (Molecular Probes, M36008, Invitrogen). The primary cultured brown adipocytes were incubated with 5 μM DHE for 30 min or 5 μM MitoSOX for 10 min and analyzed for fluorescence intensity by a fluorescent plate reader (Fluoroskan Ascent Fluorometer, Thermo LabSystems Oy) at 510 nm excitation with a 600 nm bandpass filter for DHE or at 510 nm excitation with a 580 nm bandpass filter for MitoSOX.

### Detection of ATP content

The ATP levels of primary cultured brown adipocytes were detected by ATP assay kits (Beyotime, China). Luminance was detected by a fluorescent plate reader (Fluoroskan Ascent Fluorometer, Thermo LabSystems Oy). Protein concentration was detected with a BCA protein concentration assay kit (Beyotime, China). The ATP levels were shown in nmol/mg protein.

### [Ca^2+^]_mito_ measurements

Primary cultured brown adipocytes were cultured with Rhod-2 AM (2 μmol/L, Invitrogen) for 60 min at 37 °C in intracellular medium with [Ca^2+^] as indicated. [Ca^2+^]_mito_ was monitored at 552 nm and 490/440 nm excitation wave lengths, respectively, and emission was collected at 581 nm for [Ca^2+^]_mito_.

### Patch-Clamp experiments

Mitochondria isolated from cells were placed in a hypotonic solution (5 mM HEPES, 200 μM CaCl_2_, pH = 7.2) for approximately 1 min to induce the swelling and breakage of the outer membrane. Then, a hypertonic solution (750 mM KCl, 30 mM HEPES, 200 μM CaCl_2_, pH = 7.2) was added to restore the isotonicity of the medium. The patch-clamp pipette was filled with an isotonic solution containing 150 mM KCl, 10 mM HEPES, and 200 μM CaCl_2_ at pH = 7.2. Data were recorded via a MultiClamp 700B amplifier and Digidata 1500 A converter running on Clampex 9.2 software (Axon Instruments, USA). The traces of the experiments were recorded in single-channel mode. The ionic current was measured in a symmetric 150/150 mM KCl isotonic solution with a 200 μM CaCl_2_ concentration. The mitoplasts at the tip of the measuring pipette were transferred into the openings of a larger pipe system, and their outer faces were rinsed with solutions of the channel modulators. The channel open probability (Po, open probability) was determined and presented as the mean ± SEM obtained from at least three independent experiments.

### Western blot analysis

Immunoblots of TRPV1, UCP1, PRDM16, C/EBPβ, PPARγ, PPARδ, HSL, PPARα, SREBP-1c, C/EBPα, ACC, ACC (p79), LETM1, and GAPDH were performed as described previously. Protein expression was normalized to the internal control GAPDH.

### Co-Immunoprecipitation

Immunoprecipitation (IP) was performed using ‘Cellular Labeling and IP Kits (Roche Diagnostics). Mouse BAT mitochondria were gathered in lysis buffer. IP was performed by mixing 1 µg of anti-TRPV1/UCP1/LETM1 primary antibodies (Alomone, Jerusalem, Israel/Santa Cruz Biotechnology, USA) or IgG overnight. The supernatants were incubated with 50 µl protein A agarose for 120 min to avoid nonspecial binding. Coimmunoprecipitated endogenous or overexpressed proteins were tested by immunoblotting.

### Immunofluorescence staining

Brown adipocytes were fixed with 4% paraformaldehyde and blocked with 5% bovine serum. Then, the cells were incubated with a primary antibody overnight at 4 °C and then a secondary antibody conjugated to a fluorescent probe (ZSGB-BIO, China) for 30 min. Imaging was performed by fluorescence microscopy (TE2000, Nikon, Japan).

### Measurement of temperature and locomotor activity in mice

The rectal temperature of the mice was measured by a rectal probe digital thermometer (Physitemp) inserted 1.8 cm into the colon from 9:00 AM to 10:00 AM. The core body temperature and locomotor activity were synchronously detected by the telemetry system (TA10TA–F20; Data Sciences, Inc., St. Paul, MN, USA). Mice were anesthetized, and anesthesia was maintained by halothane. The telemetric transmitter was put in the peritoneal cavity to detect core body temperature and locomotor activity [18].

### Indirect calorimetry

The O_2_ expenditure and CO_2_ generation of mice were detected in temperature-controlled metabolic chambers (Oxymax/CLAMS; Columbus Instruments, USA). The mice were placed in the chambers at 25 °C on a 12:12-h dark/light loop with free access to food and water.

### Blood pressure detection

Detected blood pressures were recorded using the tail-cuff system (BP-98A; SOFTRON Co., Tokyo, Japan). Telemetric transmitters were implanted surgically (TL11M2-C50-PXT, Data Sciences International, USA). The catheter was implanted into the descending carotid artery. The mice recovered for 10 days, and then 24-h systolic and diastolic pressures were simultaneously detected by the telemetric transmitter in conscious mice. The data were recorded for 10 s every 30 min, and the mean values were used in 24 h [19].

### Mice biochemical index detection

Serum aldosterone and angiotensin II were analyzed with a Northern immunoassay kit radioimmunoassay (Northern Institute of Biotechnology, Chima). Serum nitric oxide (NO) production was analyzed with a nitrate/nitrite colorimetric assay kit (Cayman Chemical, USA). Serum endotoxin levels were analyzed with the Pierce Chromogenic Endotoxin Quant Kit (Thermo Fisher Scientific, Waltham, MA, USA) according to the manufacturer’s instructions.

### Statistical analysis

The data are expressed as the means ± SEM from three to seven independent experiments. Comparisons between groups were analyzed by Student’s *t* test or one-way ANOVA with Bonferroni’s multiple comparison post hoc test (SPSS, Inc., Chicago, IL, USA). Two-sided *p* values less than 0.05 were considered to indicate statistical significance.

## Results

### The absence of TRPV1 exacerbated obesity and hypertension in UCP1 knockout mice

To determine how TRPV1 deficiency affects obesity induced by UCP1 knockout, we generated TRPV1/UCP1 double knockout mice (TRPV1^−/−^/UCP1^−/−^) by crossbreeding TRPV1^−/−^ mice with UCP1^−/−^ mice. Western blot results indicated that the expression of TRPV1 or UCP1 could not be detected in the BAT of the double knockout mice (Supplementary Fig. 1A, B).

**Fig. 1:**
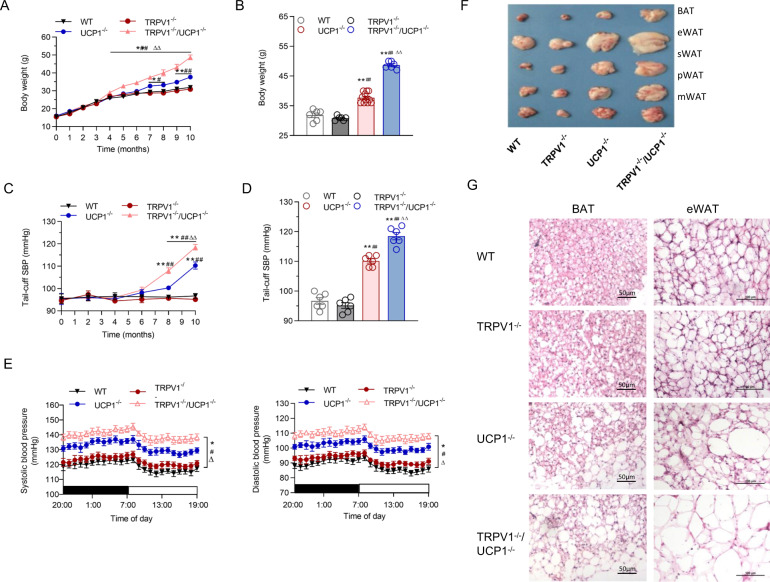
TRPV1 and UCP1 double knockout aggravated obesity and hypertension. **A**, **B** Time-dependent changes (**A**) and the 10^-^month observed (**B**) body weights in WT, TRPV1^−/−^, UCP1^−/−^ and TRPV1^−/−^/UCP1^−/−^ mice. **C** Tail-cuff systolic blood pressure (SBP) throughout the observation period. **D** Tail-cuff systolic blood pressure was detected at the 10th month of observation. **E** Twenty-four-hour ambulatory systolic (left panel) and diastolic blood pressure measurements (right panel) of the four groups of mice. **F** Photographs showing the appearances of brown and epididymal, subcutaneous, perinephritic, and mesenteric white adipose tissue (epididymal/subcutaneous/perirenal/mesenteric white adipose tissue (eWAT/sWAT/pWAT/mWAT, respectively) in WT, TRPV1^−/−^, UCP1^−/−^ and TRPV1^−/−^/UCP1^−/−^ mice at the 10th month of observation. **G** Representative images showing hematoxylin–eosin staining of BAT (left line) and eWAT (right line) in the four groups. The scale bar indicates 50–100 μm

While being fed a chow diet for 10 months, TRPV1^−/−^/UCP1^−/−^ mice developed a higher body weight than WT or TRPV1^−/−^ mice after the 4th month (*p* < 0.01). Although UCP1^−/−^ mice also rapidly became obese, the knockout of TRPV1 resulted in a more obvious body weight gain after the 7th month (*p* < 0.01). At the 10th month, the body weights of TRPV1^−/−^/UCP1^−/−^ mice were significantly higher than those of the other groups (Fig. 1A, B). The tail blood pressure of UCP1^−/−^ mice was higher than that of WT and TRPV1^−/−^ mice after the 8th month, and TRPV1 knockout further accelerated and elevated the development of hypertension in a UCP1 knockout background (Fig. 1C, D). The 24-h ambulatory arterial systolic and diastolic pressures showed consistent results (Fig. 1E).

At 10 months, the interscapular BAT of TRPV1^−/−^/UCP1^−/−^ mice was visibly paler than that of TRPV1^−/−^, UCP1^−/−^ or WT mice. The TRPV1^−/−^/UCP1^−/−^ mice appeared to be the fattest mice among the four groups of mice (Fig. 1F and Supplementary Fig. 1C). The amounts of BAT and visceral white adipose tissue (WAT) in TRPV1^−/−^/UCP1^−/−^ mice were the highest compared with those in the other three groups (Supplementary Fig. 1D, E). H&E stained sections of BAT and epididymal white adipose tissue (eWAT) revealed that the adipocytes of TRPV1^−/−^/UCP1^−/−^ mice had larger lipid droplets, indicating increased lipid content, which was unusual in the normal brown adipocytes of lean (WT or TRPV1^−/−^) mice (Fig. 1G and Supplementary Fig. 1F).

### TRPV1 knockout promoted the whitening of BAT and reduced energy expenditure in UCP1 knockout mice

**Fig. 2:**
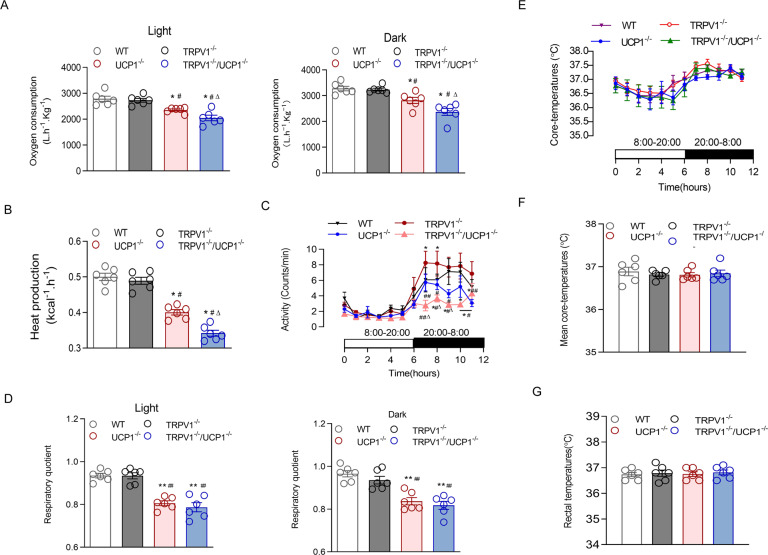
TRPV1 and UCP1 double knockout attenuated energy metabolism. **A** Oxygen consumption in the light (left panel) and dark (right panel) of WT, TRPV1^−/−^, UCP1^−/−^ and TRPV1^−/−^/UCP1^−/−^ mice was measured in metabolism cages. **B**, **C** Heat production (**B**) and 24-h locomotor activities (**C**) of the four groups of mice were measured in metabolism cages. **E**, **F** Twenty-four-hour ambulatory core body temperatures (**E**) and 24-h mean basal body temperatures (**F**) in mice were measured by the telemetry system at 25 °C ambient temperature. **G** Rectal temperatures of WT, TRPV1^−/−^, UCP1^−/−^ and TRPV1^−/−^/UCP1^−/−^ mice were measured in the anus of mice by thermometer at 8 AM every day at 25 °C ambient temperature. The values are shown as the means ± SEM; *n* = 6 per group; *P < 0.05, **P < 0.01 vs. WT mice; ^#^*P* < 0.05, ^##^*P* < 0.01 vs. TRPV1^−/−^ mice; ^∆^*P* < 0.05, ^∆∆^*P* < 0.01 vs. UCP1^−/−^ mice

We next examined the oxygen consumption of all groups of mice through indirect calorimetry. The results revealed that UCP1 knockout significantly reduced oxygen consumption, and the deficiency of TRPV1 further lowered the oxygen consumption in UCP1^−/−^ mice at any time point (Fig. 2A). Accordingly, accompanied by significantly less heat production (Fig. 2B) and lower locomotor activity (Fig. 2C) by UCP1 knockout, TRPV1 deficiency further exacerbated these effects of UCP1 knockout at certain time points even though TRPV1^−/−^ mice displayed more activity than the other groups. TRPV1 deficiency did not further aggravate the respiratory quotient (RQ) reduction in UCP1 knockout mice (Fig. 2D). However, the 24-h core body temperatures and rectal temperatures of the four groups of mice were not different at 25 °C ambient temperature (Fig. 2E–G).

In addition, the plasma triglycerides and abdominal circumference of TRPV1^−/−^/UCP1^−/−^ mice were also higher than those of UCP1^−/−^ mice or the other two groups (Supplementary Fig. 2A and Table 1). However, there was no difference in plasma total cholesterol, fasting blood glucose, or glucose tolerance, as shown by IPGTT results, among the four groups (Supplementary Fig. 2B–D). These results imply that a deficiency of TRPV1 promotes the synthesis and accumulation of lipids to induce more severe obesity and obesity-related hypertension in UCP1^−/−^ mice. The promotional effect of TRPV1 and UCP1 depletion on obesity and hypertension is associated with impaired energy expenditure and spontaneous activity, which might be a reflection of lower mitochondrial function.

### TRPV1 deficiency further reduced BAT gene expression in UCP1 knockout mice

**Fig. 3:**
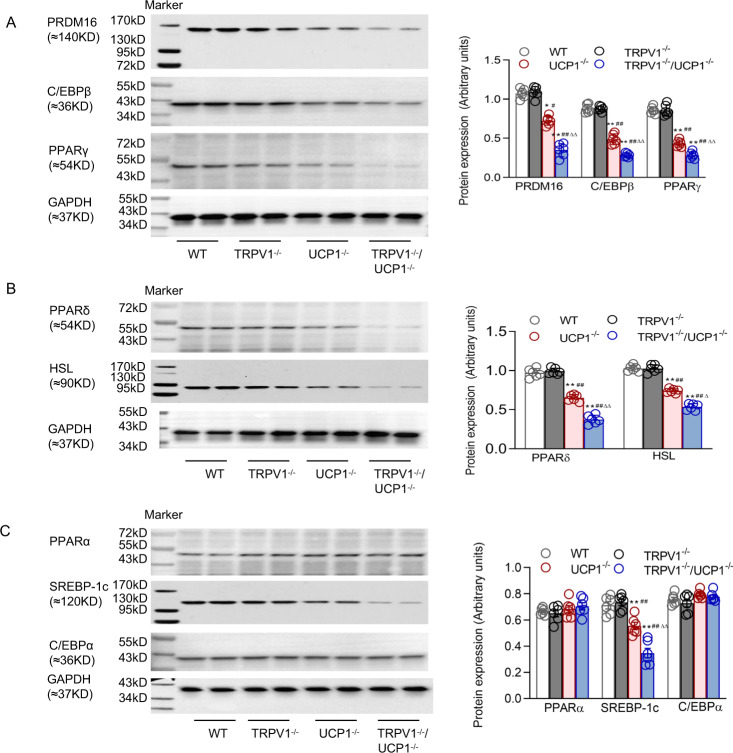
TRPV1 and UCP1 double knockout decreased functional BAT generation and lipolysis. **A**, **B**, **C** Immunoblots of PRDM16, C/EBPβ, and PPARγ (**A**); PPARδ and HSL (**B**)**;** and PPARα, SREBP-1c, and C/EBPα (**C**) in BAT from WT, TRPV1^−/−^, UCP1^−/−^ and TRPV1^−/−^/UCP1^−/−^ mice. Values are presented as the means ± SEM for six mice. *P < 0.05, **P < 0.01vs. WT mice; ^#^*P* < 0.05, ^##^*P* < 0.01 vs. TRPV1^−/−^ mice; ^∆^*P* < 0.05 vs. UCP1^−/-^ mice

To further investigate the underlying mechanism of the effects of TRPV1 knockout on BAT function, we detected the expression of some related molecules of BAT. A depletion of PRDM16 in BAT results in a loss of brown characteristics in adult mice [20]. PRDM16 and CCAAT/enhancer-binding protein beta (C/EBPβ) together are enough to trigger induced differentiation of BAT in naive fibroblastic cells [21]. We observed that the protein levels of PRDM16 and C/EBPβ in BAT were obviously decreased in TRPV1^−/−^/UCP1^−/−^ mice compared with the other groups (Fig. 3A). PPARγ is a transcription factor stimulating both the production and thermogenic activity of BAT [22]. PPARγ was remarkably suppressed in the BAT of TRPV1^−/−^/UCP1^−/−^ mice (Fig. 3A). We also examined several lipolysis-related factors, and the results showed that the expression of PPARδ and hormone-sensitive lipase (HSL) was significantly decreased in the BAT of TRPV1^−/−^/UCP1^−/−^ mice compared with that of the other groups (Fig. 3B). However, the expression levels of some lipogenesis-related factors in BAT, such as PPARα, SREBP-1c, and C/EBPα, were not altered (Fig. 3C).

It could be deduced from these results that enhanced BAT whitening is associated with reduced functional BAT generation and lipolysis in TRPV1^−^/^−^/UCP1^−/−^ mice.

### TRPV1 deficiency aggravated the mitochondrial dysfunction induced by UCP1 knockout in BAT

**Fig. 4:**
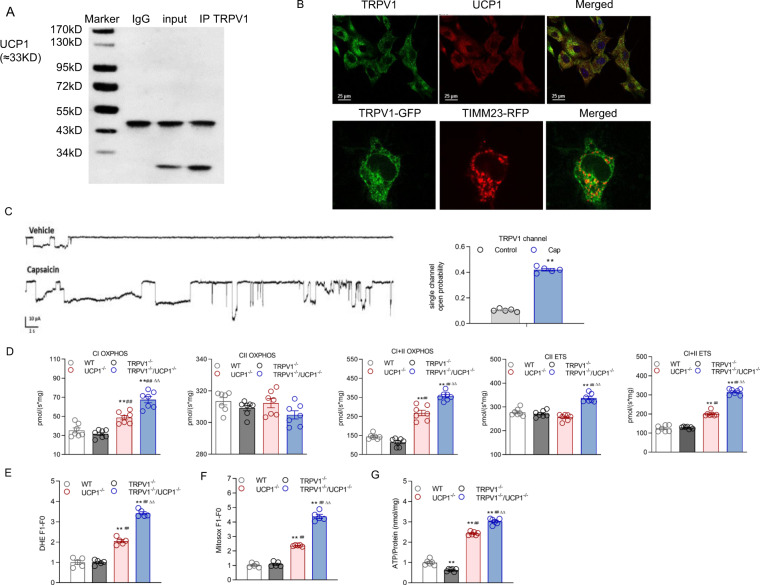
TRPV1 and UCP1 double knockout decreased functional BAT generation and lipolysis. **A** Immunoprecipitation (IP) with TRPV1 and UCP1 antibodies on mitochondria isolated from the brown adipose tissue of WT mice. The immunoblot bands are representative of six separate experiments. **B** Immunofluorescence staining of primary cultured brown adipocytes with anti-TRPV1 and UCP1 antibodies. TRPV1 was stained green, and UCP1 was stained red (upper level). Bar denotes 25 μm. The images were collected using a Nikon TE2000-U inverted fluorescence microscope. The coexpression of TRPV1 by plasmid transfection and TIMM23, a mitochondrial marker, in 293 A cells. The images were collected by a confocal microscope (A1 Nikon Co., Tokyo, Japan). Images are representative of 3 separate experiments. **C** Patch-clamp analysis of the inner membrane of the mitochondrial response to capsaicin, a TRPV1 agonist. **D** Oxygen consumption at different mitochondrial stages of primary brown adipocytes in WT, TRPV1^−/−^, UCP1^−/−^, and TRPV1^−/−^/UCP1^−/−^ mice was measured by Oxygraph-2 k high-resolution respirometry. Routine values respiration in the original state, CI_OXPHOS_ CI-dependent oxidative phosphorylation, CII_OXPHOS_ CII-dependent oxidative phosphorylation, CI + II_OXPHOS_ oxidative phosphorylation providing CI and CII substrates, CII_ETS_ noncoupled respiration with CII-dependent respiration is considered the maximum capacity of the ETS state, CI + II_ETS_ noncoupled CI and CII substrates. **E**–**G** Dihydroetorphine hydrochloride (DHE, **E**), MitoSox (**F**), and ATP (**G**) levels of primary cultured brown adipocytes were measured. *P < 0.05, **P < 0.01 vs. WT mice; ^##^*P* < 0.01 vs. TRPV1^−/−^ mice; ^∆∆^*P* < 0.01 vs. UCP1^−/−^ mice

Mitochondrial dysfunction and oxidative injury are considered crucial elements in cell metabolism and pathophysiological processes. Thus, we explored whether TRPV1 and UCP1 double knockout affect the mitochondrial functions of BAT. First, the immunoprecipitation results of mitochondria from BAT showed that there was an obvious interaction between TRPV1 and UCP1 in WT mice (Fig. 4A). Furthermore, we observed that TRPV1 and UCP1 were colocalized in mouse brown adipocytes by immunofluorescent staining (Fig. 4B). Through exogenous overexpression of TRPV1 in 293 A cells by plasmid transfection, TRPV1 colocalized with TIMM23, a mitochondrial marker (Fig. 4B). Patch-clamp analysis revealed an increased current in the inner membrane of the mitochondria-attached model in response to treatment with capsaicin, a TRPV1 agonist (Fig. 4C). These results indicated that TRPV1 was localized in and functioned as a cation channel in mitochondria.

We further examined mitochondrial function in BAT isolated from mice using high-resolution respirometry. Compared with mitochondria from BAT of WT or TRPV1^−/−^ mice, mitochondria from BAT of UCP1^−/−^ mice displayed remarkably impaired mitochondrial function, including oxidative phosphorylation and electron transport capability of complex I. These indices were further increased in TRPV1^−/−^/UCP1^−/−^ mice (Fig. 4D). However, none of the complex II-related parameters were altered by TRPV1 or UCP1 knockout (Fig. 4D).

In addition, the analysis of ATP content in primary cultured brown adipocytes revealed that TRPV1 deficiency exacerbated ATP accumulation in BAT induced by UCP1 knockout adipocytes (Fig. 4E). Similarly, a loss of TRPV1 also led to more active ROS generation in the mitochondria of brown adipocytes from UCP1^−/−^ mice, as demonstrated by DHE and MitoSox fluorescent staining (Fig. 4F, G).

These results indicated that TRPV1 absence aggravated the oxidative stress generated by UCP1 deficiency.

### TRPV1 deficiency aggravated mitochondrial Ca^2+^ overload through the upregulation of LETM1

Mitochondria are the main responders and modulators of cytosolic [Ca^2+^]. Ca^2+^-mediated signals passing through the IMM are associated with an elevated ATP generation rate because Ca^2+^ regulates essential metabolic enzymes and transporters [12].

**Fig. 5:**
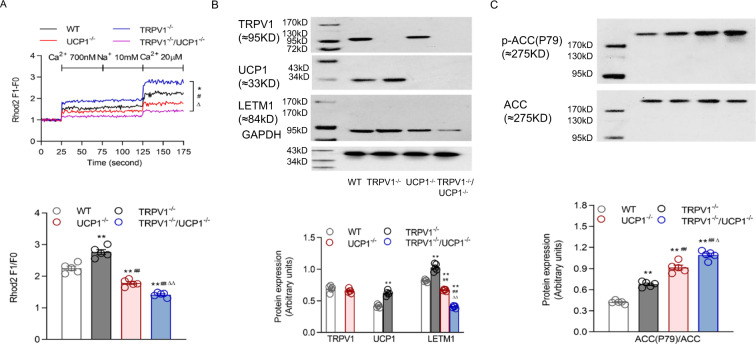
TRPV1 and UCP1 double knockout impaired mitochondrial ion balance. **A** Mitochondrial [Ca^2+^] of primary cultures of brown adipocytes from WT, TRPV1^−/−^, UCP1^−/−^ and TRPV1^−/−^/UCP1^−/−^ mice was measured after loading with Rhod2 (2 μmol/L). The lower panel is the bar graph derived from the upper figure with [Ca^2+^] at 20 μM. **B**, **C** Immunoblots of TRPV1, UCP1, and LETM1 (**B**)**;** ACC (P79); and ACC (**C**) in the BAT of WT, TRPV1^−/−^, UCP1^−/−^ and TRPV1^−/−^/UCP1^−/−^ mice. Values are presented as the means ± SEM for five experiments (the third row corresponds to the panel of the second row). *P < 0.05, **P < 0.01 vs. WT mice; ^#^*P* < 0.05, ^##^*P* < 0.01 vs. TRPV1^−/−^ mice; ^∆^*P* < 0.05, ^∆∆^*P* < 0.01 vs. UCP1^−/−^ mice

Thus, we detected Ca^2+^ uptake in mouse primary brown adipocytes and observed that the cells from TRPV1^−/−^/UCP1^−/−^ mice displayed obviously decreased [Ca^2+^]_mito_ uptake when [Ca^2+^]_cyto_ was low (Fig. 5A). Usually, the Na^+^/Ca^2+^ exchanger regulates the homeostasis of [Ca^2+^]_mito_ with low [Ca^2+^]_cyto_ through Na^+^-dependent Ca^2+^_mito_ extrusion. However, the decreased steady-state [Ca^2+^]_mito_ remained constant in the brown adipocytes from TRPV1^−/−^/UCP1^−/−^ mice in response to 10 mM Na^+^. Conversely, high [Ca^2+^]_cyto_ promoted a rapid increase in the [Ca^2+^]_mito_ of the brown adipocytes from TRPV1^−/−^ mice to a higher level than those of the other three groups (Fig. 5A). However, the loss of TRPV1 further reduced Ca^2+^_mito_ uptake in the UCP1 knockout background (Fig. 5A). These results indicate that TRPV1 deficiency further lowered the reduced Ca^2+^ intake by UCP1 knockout in mitochondria.

Consistently, the expression levels of both UCP1 and LETM1 were compromised and upregulated in the BAT of TRPV1^−/−^ mice. UCP1 knockout led to a significant decrease in LETM1 expression in mouse BAT, which was further reduced in the absence of TRPV1, suggesting that the inhibition of LETM1 expression by TRPV1 was a critical mechanism maintaining Ca^2+^ homeostasis and that this mechanism disappeared upon UCP1 loss (Fig. 5B). Accordingly, acetyl coenzyme A carboxylase (ACC) triggers the generation of long-chain fatty acids, reflecting the conversion of ATP to fat in BAT. We found that the phosphorylation of ACC was upregulated in the BAT of UCP1^−/−^ mice and was further heightened in TRPV1^−/−^/UCP1^−/−^ mice (Fig. 5C).

### TRPV1, UCP1 and LETM1 act as a complex to maintain mitochondrial Ca^2+^ homeostasis in BAT

**Fig. 6:**
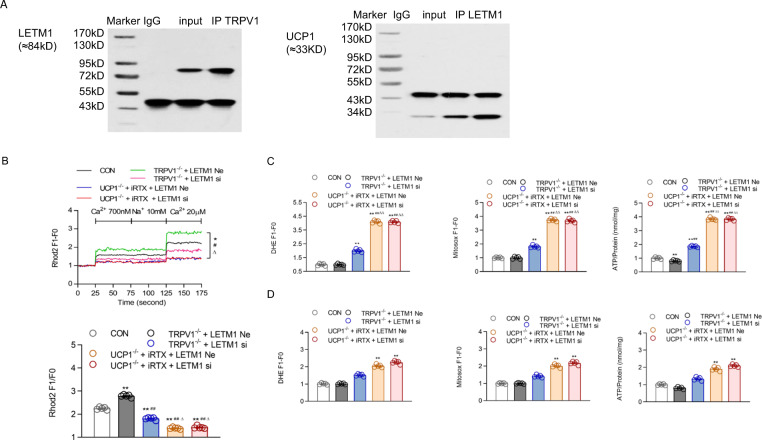
Mechanism by which TRPV1 and UCP1 double knockout cause mitochondrial ion imbalance. **A** Immunoprecipitation (IP) with TRPV1/LETM1 and LETM1/UCP1 antibodies on mitochondria isolated from the brown adipose tissue of WT mice. The immunoblot bands are representative of six separate experiments. **B** Mitochondrial [Ca^2+^] of primary cultures of brown adipocytes from WT, TRPV1^−/−^ and UCP1^−/−^ mice, as well as TRPV1^−/−^ and UCP1^−/−^ mice transfected with LETM1 siRNA, was measured after loading with Rhod2 (2 μmol/L). The lower panel is the bar graph derived from the left figure with [Ca^2+^] at 20 μM. **C**, **D** Dihydroetorphine hydrochloride (DHE, **C**/**D**, left panel) and ATP (**C**/**D**, right panel) levels of primary cultured brown adipocytes were measured before (**C**) and after tempol treatment (200 µM, **D**) treatment. MitoSox levels of primary cultured brown adipocytes were measured before (**C**, middle panel) and after mito-tempo (20 µM, **D**, middle panel) treatment. *P < 0.05, **P < 0.01 vs. WT mice (CON); ^##^*P* < 0.01 vs. TRPV1^−/−^ mice + LETM1 siRNA negative control (TRPV1^−/−^ + LETM1 Ne); ^∆∆^*P* < 0.01 vs. UCP1^−/−^ mice + iRTX + LETM1 siRNA negative control (UCP1^−/−^ + LETM1 Ne)

Immunoprecipitation in the mitochondria of BAT showed that both TRPV1 and UCP1 bind to LETM1; thus, these three molecules formed a complex (Fig. 6A). The knockout of TRPV1 resulted in a significant increase in exogenous Ca^2+^ influx into mitochondria, and LETM1 knockdown reduced the [Ca^2+^]_mito_ increase in the primary brown adipocytes of TRPV1^−/−^ mice, indicating that the higher expression of LETM1 was the main reason for the high mitochondrial Ca^2+^ uptake when TRPV1 was absent (Fig. 6B). In addition, when UCP1 was knocked out, this compensatory response of LETM1-mediated Ca^2+^ influx was completely blocked, resulting in a very low mitochondrial Ca^2+^ influx (Fig. 6B).

Accordingly, mitochondrial ROS production and ATP biosynthesis were also remarkably elevated in brown adipocytes from UCP1 knockout mice treated with iRTX, an inhibitor of TRPV1 (Fig. 6C). The promotional effect of LETM1 siRNA on ROS and ATP production in TRPV1 knockout brown adipocytes was completely blocked by UCP1 knockout, as the two parameters were almost equal with or without si-LETM1 (Fig. 6C). These findings imply that the knockout of TRPV1 alone might lead to a compensatory response of LETM1 overexpression to increase mitochondrial Ca^2+^ uptake to maintain Ca^2+^ homeostasis and reduce ROS production. This response was dependent on the existence of UCP1, as the knockout of UCP1 completely erased this response and augmented ROS production in BAT. However, after treatment with tempol or Mito-TEMPO, an ROS neutralizer, the effects of TRPV1/UCP1 double knockout or LETM1 knockdown were counteracted (Fig. 6D). This indicated that the ROS production mediated by [Ca^2+^]_mito_ disorder was increased with TRPV1/UCP1 double knockout or LETM1 knockdown, which could be reduced by an ROS neutralizer.

### Activation of the RAAS and ROS production contributed to obesity-induced hypertension in TRPV1/UCP-1 double knockout mice

**Fig. 7:**
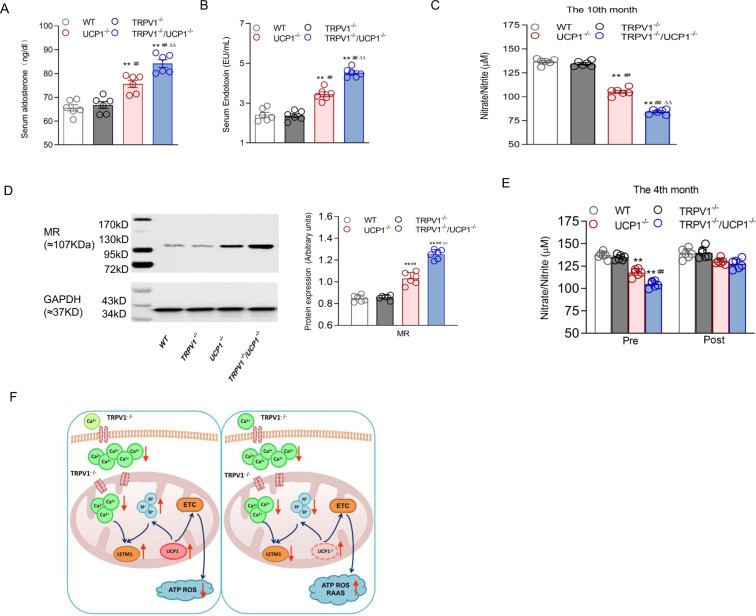
Elevated blood pressure induced by TRPV1 knockout synergizing with UCP1 knockout through the activation of the RAA system and ROS production. **A**–**C** Serum aldosterone (**A**), endotoxin (**B**) and NO (**C**) were detected in WT, TRPV1^−/−^, UCP1^−/−^ and TRPV1^−/−^/UCP1^−/−^ mice. **D** Immunoblots of mineralocorticoid receptors (MRs) in the white adipose tissue of WT, TRPV1^−/−^, UCP1^−/−^ and TRPV1^−/−^/UCP1^−/−^ mice. **E** The pre- and post-tempol (3 mmol/L in drinking water)-treated serum NO of WT, TRPV1^−/−^ and UCP1^−/−^ mice. **F** The working model for the deficiency of TRPV1 (left) and TRPV1&UCP1 double knockout (right) in brown adipose tissue causes obesity and the associated hypertension. TRPV1 knockout aggravates the dysfunction of [Ca^2+^]_mito_ uptake caused by UCP1 knockout, increases the production of ATP and ROS, activates the RAA system, and leads to higher body weight and blood pressure. Values are presented as the means ± SEM for six experiments. *P < 0.05, **P < 0.01 vs. WT mice; ^#^*P* < 0.05, ^##^*P* < 0.01 vs. TRPV1^−/−^ mice; ^∆^*P* < 0.05, ^∆∆^*P* < 0.01 vs. UCP1^−/−^ mice

To further explore whether the activation of the RAAS was involved in the promotional effect of TRPV1 knockout, we first measured the serum concentrations of aldosterone. Compared to the other three groups, the serum aldosterone and endotoxin levels were increased and the serum NO level was decreased in TRPV1^−/−^/UCP1^−/−^ mice (Fig. 7A–C). Accordingly, mineralocorticoid receptors (MRs) in white adipose tissues were mostly activated in TRPV1^−/−^/UCP1^−/−^ mice (Fig. 7D).

To further determine the importance of ROS production in the promotional effect of TRPV1 knockout on obesity-related hypertension induced by UCP1 knockout, we added tempol, an ROS scavenger, to the feed of TRPV1/UCP1 double knockout mice. The addition of tempol significantly increased serum NO in double knockout mice to a level equal to that of WT mice, completely erasing the effect of either TRPV1 or UCP1 knockout (Fig. 7E), indicating that increased ROS production was a key factor that resulted in the development of hypertension in the double knockout mice.

According to these results, TRPV1 deficiency may aggravate the elevated blood pressure caused by UCP1 ablation by activating the RAA system and the accompanying oxidative stress.

## Discussion

The combination of obesity and hypertension not only increases the risk of refractory arterial hypertension, such as renal sympathetic denervation [23], but also increases the incidence rate and mortality of cardiovascular diseases, including coronary heart disease, sudden cardiac death, chronic kidney disease, and stroke. In this study, we show that the promotional effect of TRPV1 deficiency on BAT whitening and obesity induced by UCP1 knockout relies on aggravated mitochondrial Ca^2+^ disorder and ROS production. Elevated ROS production induced by TRPV1 knockout synergized with elevated blood pressure induced by UCP1 knockout through the activation of the RAA system. Mechanistically, TRPV1 knockout further induced mitochondrial dysfunction in BAT by repressing LETM1 expression, which increased mitochondrial calcium uptake in UCP1 knockout mice (Fig. 7F).

The exact pathophysiological mechanism linking weight changes with blood pressure changes has not been fully explored. Based on current studies, obesity-related hypertension occurs via multiple mechanisms: insulin resistance; adipokine alterations as well as related inflammatory and oxidative reactions; inappropriate SNS and RAAS activation; structural and functional abnormalities in the kidney, heart, and vasculature; and maladaptive immunity [24]. These neurohormonal, renal, and inflammatory mechanisms of obesity-related hypertension are interdependent. Abnormal activation of the RAAS increases ROS production and oxidative stress [25]. This study showed that the occurrence of obesity-associated hypertension was directly related to the decline in mitochondrial function in BAT depending on ROS production. ROS production causes oxidative stress, which contributes to vascular dysfunction and hypertension. The inhibition of ROS production can prevent the occurrence of hypertension [26]. Mitochondria are the most important organelles for the production of reactive oxygen species. In the past, there was limited understanding of the important role of mitochondrial dysfunction in obesity-related hypertension. In this study, we found that TRPV1 knockout alone did not increase ROS production or the occurrence of hypertension in WT mice, but the knockout of TRPV1 further exacerbated ROS production in the BAT of UCP1 knockout mice. Furthermore, the knockdown of LETM1 blocked the elevated mitochondrial Ca^2+^ uptake induced by TRPV1 knockout, indicating that a compensatory response existed between TRPV1 and LETM1, and the upregulation of LETM1 after TRPV1 knockout maintained normal mitochondrial function. The knockdown of LETM1 hampered this mechanism, resulting in increased ROS and ATP production. The compensatory mechanism disappeared after UCP1 knockout, indicating the necessity of UCP1 in the maintenance of the complex containing TRPV1, LETM1, and UCP1, which was determined by Co-IP in this study. However, the detailed mechanism underlying the regulatory effect of UCP1 on TRPV1 or LETM1 needs to be further investigated. These results highlight a critical molecular mechanism maintaining the appropriate mitochondrial Ca^2+^ level in BAT that reduces obesity-related hypertension.

Obesity is associated with RAAS overactivation, as demonstrated by increased adipose tissue MR expression [27] and elevated plasma levels of aldosterone, renin, angiotensinogen, angiotensin-converting enzyme (ACE), and AT II [28]. Increased levels of aldosterone act on adipose tissue MRs to increase lipid storage, release proinflammatory adipokines, and suppress BAT thermogenesis [29, 30]. Accordingly, as ROS mainly affect aldosterone production, serum aldosterone and endotoxin levels were high in TRPV1^−/−^/UCP1^−/−^ mice, accompanied by decreased serum NO, increased oxidative stress production, and enhanced RAAS activation.

Ca^2+^-mediated signaling passing through the inner membrane of mitochondria is associated with an elevated ATP generation rate because Ca^2+^ regulates essential metabolic enzymes and transporters [31]. Generally, free mitochondrial [Ca^2+^] varies in a very small range in living cells. This means that Ca^2+^ exchangers are critical to keep steady-state mitochondrial Ca^2+^ at a low cytoplasmic Ca^2+^ to conserve mitochondrial homeostasis and bioenergy by mediating Ca^2+^ passage through the inner mitochondrial membrane [32]. LETM1 encodes a mitochondrial Ca^2+^/H^+^ exchanger. Decreased mitochondrial Ca^2+^ uptake led to impaired ATP synthesis capability when Letm1 was silenced in cells [14]. LETM1 impairs insulin signaling and the PI3K/PKB pathway in the adipose tissue of obese mice, and its expression is reduced in obesity [15]. The present study revealed a previously unrecognized role of LETM1 in obesity-related hypertension by acting as a mitochondrial Ca^2+^ modulator in BAT. In addition, LETM1 is also required for the browning of WAT to form beige adipocytes, indicating a promotional effect of LETM1 in the maintenance of BAT [33]. In our study, we observed that the knockout of TRPV1 directly upregulated the expression of both UCP1 and LETM1, suggesting that TRPV1 is involved in controlling the normal activity of BAT, which might be related to its regulatory effect on sympathetic nerves, as the activation of TRPV1 in the nucleus tractus solitarius has been reported to inhibit BAT sympathetic nerve activity and decrease BAT metabolism [34]. Consistently, our previous report also indicated that the inhibitory effect of TRPV1 on diet-induced obesity required higher BAT activity, which might affect the whole-body metabolic rate [35]. Therefore, an appropriate mitochondrial Ca^2+^ level is a key factor that maintains normal BAT structure and function, in which the regulatory effect of TRPV1 might play a critical role.

## Conclusion

In summary, the knockout of TRPV1 and UCP1 in mice at the same time induced severe obesity and obesity-associated hypertension. The results of this study showed that Letm1-mediated mitochondrial calcium uptake might be a compensatory mechanism accounting for the ion homeostasis of BAT mitochondria after TRPV1 knockout. However, in UCP1 knockout mice, when the inhibitory effect of TRPV1 on LETM1 disappeared, the knockout of TRPV1 reversely aggravated mitochondrial calcium disorder and subsequent ROS production in BAT, which led to the development of hypertension. Thus, the regulation of mitochondrial Ca^2+^ homeostasis and physiological function may represent a new therapeutic target for obesity and obesity-associated hypertension.

## Supplementary information

Supplementary Figures

Supplementary Table
