# Supplementary figures and images for: Lack of TRPV1 aggravates obesity-associated hypertension through the disturbance of mitochondrial Ca2+ homeostasis in brown adipose tissue
Source: Hypertens Res. 2022 Jan 18;45(5):789–801. doi: 10.1038/s41440-021-00842-8 (PMC9010289; doi:10.1038/s41440-021-00842-8)

Supplementary Figure 1

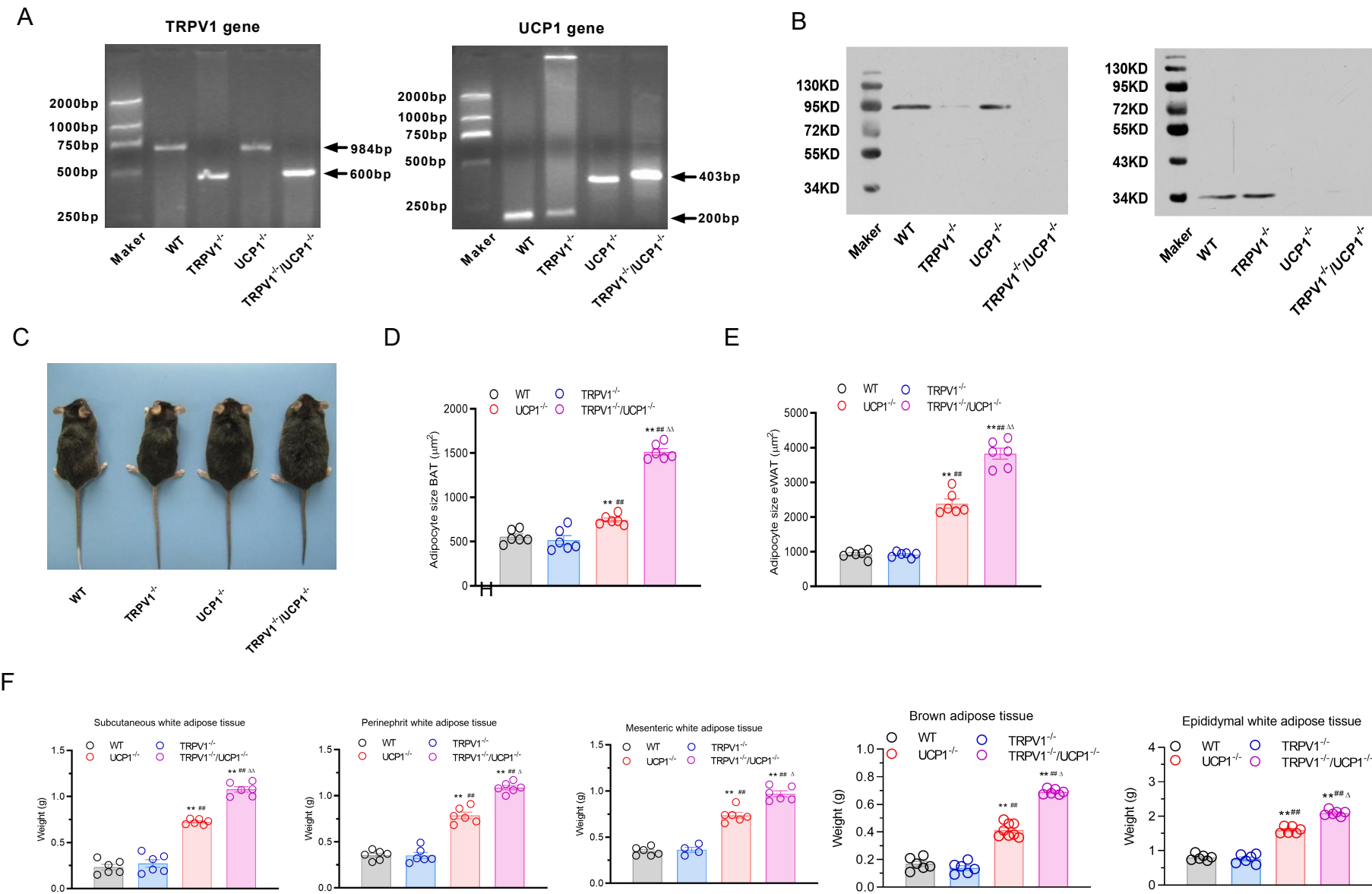

Supplementary Figure 2

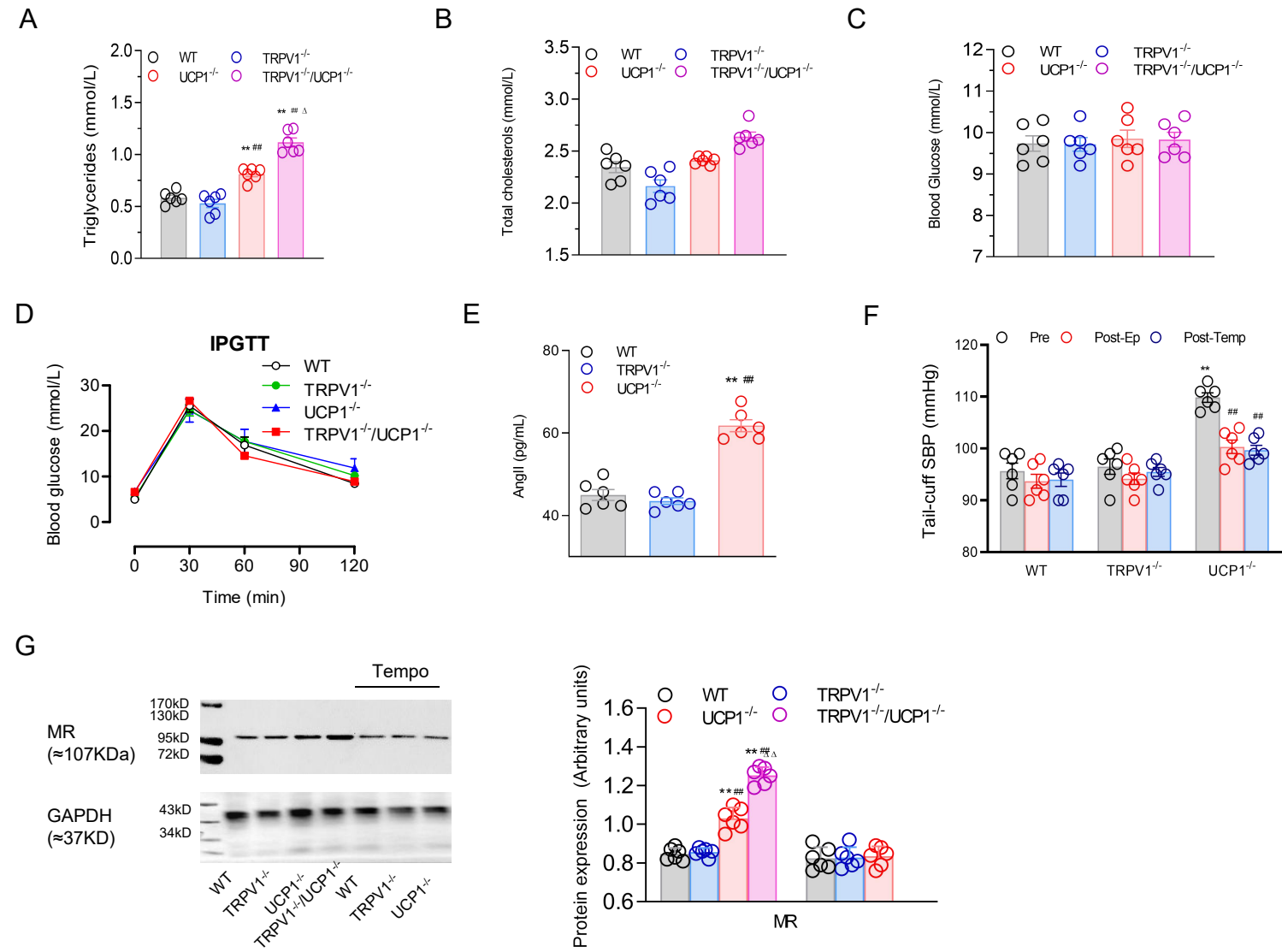

Supplement: Supplementary file 1 — Supplementary Figures [file 41440_2021_842_MOESM1_ESM.pdf]
