# Supplementary material for: Lack of TRPV1 aggravates obesity-associated hypertension through the disturbance of mitochondrial Ca2+ homeostasis in brown adipose tissue
Source: Hypertens Res. 2022 Jan 18;45(5):789–801. doi: 10.1038/s41440-021-00842-8 (PMC9010289; doi:10.1038/s41440-021-00842-8)
Supplement: Supplementary file 2 — Supplementary Table [file 41440_2021_842_MOESM2_ESM.docx]

|  | WT | TRPV1^-/-^ | UCP1^-/-^ | TRPV1^-/-^/  UCP1^-/-^ |
| --- | --- | --- | --- | --- |
| Food intake  (the fist 10 days, g/day·animal) | 4.43±0.03 | 4.44±0.05 | 4.57±0.07 | 4.55±0.09 |
| Food intake  (the last 10 days, g/day·animal) | 4.39±0.09 | 4.39±0.21 | 4.19±0.11 | 4.39±0.26 |
| HDL-c (mmol/L) | 1.37±0.10 | 1.28±0.08 | 1.45±0.16 | 1.48±0.10 |
| LDL-c (mmol/L) | 0.21±0.01 | 0.22±0.01 | 0.20±0.02 | 0.21±0.02 |
| Abdominal circumference(cm) | 6.20±0.18 | 6.17±0.24 | 10.65±0.19^**##^ | 11.85±0.23^**##ΔΔ^ |

The values were shown as mean ± SEM; n=6 per group; **P＜0.01 vs. WT mice; ^##^P＜0.01 vs. TRPV1^-/-^ mice; ^∆∆^P＜0.01 vs.UCP1^-/-^ mice.
